# Etanercept prevents TNF-α mediated mandibular bone loss in *FcγRIIb^-/-^* lupus model

**DOI:** 10.1371/journal.pone.0250215

**Published:** 2021-04-16

**Authors:** Nithidol Sakunrangsit, Piyanuch Metheepakornchai, Sarinya Kumpunya, Matthew Blake Greenblatt, Asada Leelahavanichkul, Prapaporn Pisitkun, Sutada Lotinun

**Affiliations:** 1 Skeletal Disorders Research Unit, Department of Physiology, Faculty of Dentistry, Chulalongkorn University, Bangkok, Thailand; 2 Interdisciplinary Program of Biomedical Sciences, Graduate School, Chulalongkorn University, Bangkok, Thailand; 3 Department of Pathology and Laboratory Medicine, Weill Cornell Medicine, Research Division, Hospital for Special Surgery, New York, NY, United States of America; 4 Division of Immunology, Department of Microbiology, Faculty of Medicine, Chulalongkorn University, Bangkok, Thailand; 5 Division of Allergy, Immunology, and Rheumatology, Department of Medicine, Faculty of Medicine, Ramathibodi Hospital, Mahidol University, Bangkok, Thailand; University of Vermont, UNITED STATES

## Abstract

Patients with systemic lupus erythematosus are at increased risk for alveolar bone loss due to periodontitis possibly as a result of a pathogenic immune response to oral bacteria and inflammation. The aim of the present study was to investigate whether an anti-TNF-α antagonist could prevent mandibular bone loss in the *FcγRIIb*^*-/-*^ mouse model of lupus. Mice lacking *FcγRIIb* had decreased cancellous and cortical bone volume at 6 months of age. Etanercept increased cancellous but not cortical bone volume in WT and increased both cancellous bone volume and cortical thickness in *FcγRIIb-*deficient mice. *FcγRIIb* deficiency decreased mRNA levels for osteoblast marker genes, *Osx*, *Col1a1* and *Alp* without any change in osteoclast marker genes. Etanercept increased *Osx*, *Alp*, and *Ocn* in both WT and *FcγRIIb*^*-/-*^ mice. Osteoclast marker genes including *TNF-*α, *Trap* and *RANKL/OPG* ratio was decreased in WT. Serum markers of proinflammatory cytokines, TNF-α, IFNγ, IL-6, and IL-17A, were increased in *FcγRIIb*^*-/-*^ mice and etanercept antagonized these effects in *FcγRIIb*^*-/-*^ mice. Etanercept increased serum PTH levels in the *FcγRIIb*^*-/-*^ mouse model of lupus. Our results suggest that deletion of *FcγRIIb* induces osteopenia by increasing the level of proinflammatory cytokines. Etanercept is effective in preventing mandibular bone loss in *FcγRIIb*^*-/-*^ mice, suggesting that anti-TNF-α therapy may be able to ameliorate mandibular bone loss in SLE patients with periodontitis.

## Introduction

Periodontitis is a chronic inflammation and destruction of periodontal tissue leading to mandibular alveolar bone loss induced by osteoclasts. Gram-negative bacteria, including *Porphyromonas gingivalis* and *Aggregatibacter actinomycetemcomitans* were identified as major periodontal pathogens [1]. They produce virulence factors that disturb host-microbe homeostasis. In addition to the microbial challenge, the progression of periodontitis is caused by local inflammation and over activation of the host immune response which stimulates osteoclast activity leading to alveolar bone loss [2]. Myeloid cells including monocytes and macrophages may be responsible for collateral damage to the periodontal tissues. Phagocytosis of pathogenic microorganisms by myeloid cell populations leads to the penetration of bacteria into periodontal tissue [3].

Immunoglobulin Fc receptors (FcRs) are expressed on a wide range of cells and mediate recognition of the Fc region of immunoglobulin. Fc gamma receptor (FcγR) play crucial roles in antibody-mediated immune responses. Four classes of FcγR, FcγRI, FcγRII, FcγRIII and FcγRIV have been identified in mammals [4]. Binding of immune complexes to FcγR results in phagocytosis of IgG-opsonized particles, antibody-dependent cellular cytotoxicity, and release of inflammatory mediators. During periodontal infection, polymorphonuclear neutrophilic leukocytes (PMN) expressing FcγRIIa are involved in periodontal tissue degradation by releasing reactive oxygen species and proteases. It has been shown that periodontitis patients with FcγRIIa-131H/H genotype have hyper-reactivity in response to stimulation with pathogenic bacteria leading to more severe periodontal breakdown and bone loss than the patients with FcγRIIa‐131H/R or 131R/R genotype [5].

A potential correlation between periodontal and autoimmune diseases, including systemic lupus erythematosus (SLE) has been shown. Approximately 93.8% of SLE patients had periodontitis [6]. SLE is a chronic autoimmune disease characterized by the loss of B and T cell tolerance to self-antigens, resulting in inflammation in various part of the body. SLE patients are at increased risk for periodontitis possibly as a result of a pathogenic immune response to oral bacteria and inflammation. The pathogenesis of abnormal inflammation in SLE is not completely understood. FcγRIIb, a negative regulator of B cell receptor signaling, is associated with SLE. Mice deficient in *FcγRIIb* exhibit SLE and its partial restoration rescues the disease [7, 8]. *FcγRIIb* deficiency leads to decreased disposal of immune complexes, the breakdown of self-tolerance and inability to modulate inflammatory response. *FcγRIIb*-deficient H-2^b^ mice are prone to collagen-induced arthritis [9]. Destructive cartilage and focal eroded bone surface are found in histological examination of arthritic paws.

Using anti-dsDNA antibody, a marker of SLE, our previous study indicated that mice deficient in *FcγRIIb* developed spontaneous SLE at 6 months of age. It has been reported that SLE patients have multiple B cell abnormalities, including an increase in the number of circulating plasma cells [10]. These patients produce a variety of autoantibodies directed against nuclear, cytoplasmic and cell surface autoantigens. The SLE disease activity correlates with the frequency of circulating plasma cells [11]. Our flow cytometry confirmed that B220^low^CD138^+^ plasma cells, were increased in 6 but not 3 months old *FcγRIIb* ^*-/-*^ mice [12]. *FcγRIIb*^*-/-*^ mice were osteopenic in both cortical bone and cancellous bone in tibiae. Cortical bone area and mechanical properties were reduced at 6 months of age. Deletion of *FcγRIIb* induced cancellous bone loss in tibiae due to increased bone resorption without any change in bone formation. *FcγRIIb*-deficient mice displayed increased serum levels of the proinflammatory cytokine, tumor necrosis factor-alpha (TNF-α). Therefore, deletion of *FcγRIIb* increased TNF-α-mediated bone resorption leading to inflammatory bone loss. However, the mechanisms by which absence of *FcγRIIb* affects mandibular bone turnover during inflammation have not been elucidated. Patients with periodontitis have higher serum and saliva levels of TNF-α than healthy individuals [13]. *FcγRIIa* and *FcγRIIb* polymorphism are associated with SLE and periodontitis. SLE patients who have the combined *FcγRIIa-R131* and *FcγRIIb-232T* alleles exhibit more severe tissue destruction compared to other SLE patients [14].

The present study aimed to investigate whether administration of an anti-TNF-α inhibitor could ameliorate mandibular bone loss in *FcγRIIb*^*-/-*^ mice. Similar to observations in long bones, deletion of *FcγRIIb* induced mandibular bone loss in 6 months old mice. Etanercept (Enbrel), a soluble TNF p75 receptor that acts as a TNF-α antagonist by inhibiting TNF-α interaction with its cell surface receptor, significantly decreased systemic inflammation and bone loss in the *FcγRIIb*^*-/-*^ lupus model. Anti-TNF-α increased serum PTH level only in *FcγRIIb*^*-/-*^ mice. Our results suggested that absence of *FcγRIIb* induces inflammation and mandibular bone loss and that the anti-TNF-α antagonists ameliorate these effects.

## Materials and methods

### Animals

*FcγRIIb*^-/-^ mice were provided by Dr. Silvia Bolland (NIAID, NIH, Maryland, USA) and housed at the Faculty of Medicine, Chulalongkorn University. All animal procedures were approved by the Institutional Animal Care and Use Committee at Faculty of Medicine, Chulalongkorn University. Mice were maintained in accordance with the Guide for the Care and Use of Laboratory Animals (eighth edition), National Research Council. They had free access to water and standard rodent chow (C.P. Mice Feed, Perfect Companion Group Co., Ltd., Thailand)

Male and female heterozygous (*FcγRIIb*^+/-^) mice were crossed to generate *FcγRIIb*^-/-^ mice and their WT littermates. *FcγRIIb*^-/-^ and WT controls were genotyped by PCR from tail biopsies as described earlier [15]. Three- and 6-month-old *FcγRIIb*^-/-^ males and WT controls were used to evaluate a skeletal phenotype. They were anesthetized with isoflurane and sacrificed by cervical dislocation. Left mandibles were fixed in 10% neutral buffered formalin for microcomputed tomography (μCT) analysis.

### Anti-TNF-α treatment

For etanercept studies, 6-month-old *FcγRIIb*^-/-^ males and WT controls were subcutaneously injected with 16 doses of either PBS or 25 mg/kg etanercept (Enbrel, Wyeth, New Jersy, USA) which is an intermediate dose twice a week for 8 weeks [16]. At the end of the experiment, blood samples were collected, centrifuged at 10,000 rpm for 10 minutes and kept at −80°C for determination of serum proinflammatory cytokines and PTH levels. Left mandibles were fixed in 10% neutral buffered formalin for μCT analysis. Right mandibles were frozen in liquid nitrogen and kept at −80°C for RNA isolation and qPCR.

### μCT analysis

μCT was used to analyze cortical and cancellous bone microarchitecture using a desktop μCT35, (Scanco Medical, Basserdorf, Switzerland) in accordance with recommended guidelines [17]. Buccal-lingual cross slices of the first mandibular molars were scanned at 7 μm isotropic voxel size, 73 kVp, and 113 μA. Mandibular bone scans were subjected to Gaussian filtration and segmentation and the threshold was set at 33% of the maximal gray scale value. For cortical bone, cross-sectional volume (mm^3^), cortical volume (mm^3^), marrow volume (mm^3^), cortical thickness (mm), and bone mineral density (BMD, mgHA/cm^2^) were determined. Cancellous bone volume (BV/TV, %), trabecular thickness (Tb.Th, mm), trabecular number (Tb.N, /mm), trabecular separation (Tb.Sp, mm), structure model index (SMI), and bone mineral density (mgHA/cm^2^) were analyzed.

### Quantitative real-time PCR (qPCR) analysis

Total RNA was extracted from the right mandibles using Trizol reagent (Invitrogen, Carlsbad, CA, USA) following the manufacturer’s procedure. RNA samples were further purified using an RNeasy Mini kit (Qiagen, Germantown, MD, USA) and the RNA yields were measured using a NanoDrop 1000 (Thermo Fisher Scientific, CA, USA). The cDNA was synthesized from 500 ng of total RNA with SuperScript VILO cDNA synthesis kit (Invitrogen, Carlsbad, CA, USA). The qPCR reaction was performed using the Luna Universal qPCR Master Mix (New England Biolabs) and was conducted at 60°C for 40 cycles using CFX96^TM^ Optics Module (Bio-Rad, CA, USA). Gene expression profiles were normalized to GAPDH. The oligonucleotide primers for qPCR are shown in Supplementary S1 Table.

### Serum chemistry

Mouse serum IL-23, IL1α, TNF-α, IFNγ, MCP-1, IL-12p70, IL-1β, IL-10, IL-6, IL-27, IL-17A, IFNβ, and GM-CSF levels were analyzed using a multiplex beads-based assay (LEGENDplex^TM^) according to the manufacturer’s instructions (BioLegend, San Diego, CA, USA). Serum PTH was measured using the mouse PTH (1–84) ELISA kit (Quidel, San Diego, CA) that detects the biologically active intact form of PTH.

### Statistical analysis

All results are presented as mean ± SEM. Unpaired Student’s *t*-tests were used to compare two group means. Multiple comparisons were analyzed by one-way ANOVA followed by Fisher’s protected least significant difference test. Interactions between etanercept and *FcγRIIb* deficiency were analyzed by two-way ANOVA. Differences were considered statistically significant at *p* < 0.05.

## Results

### Deletion of *FcγRIIb* induced osteopenia in the mandible by 6 months of age

**Fig 1:**
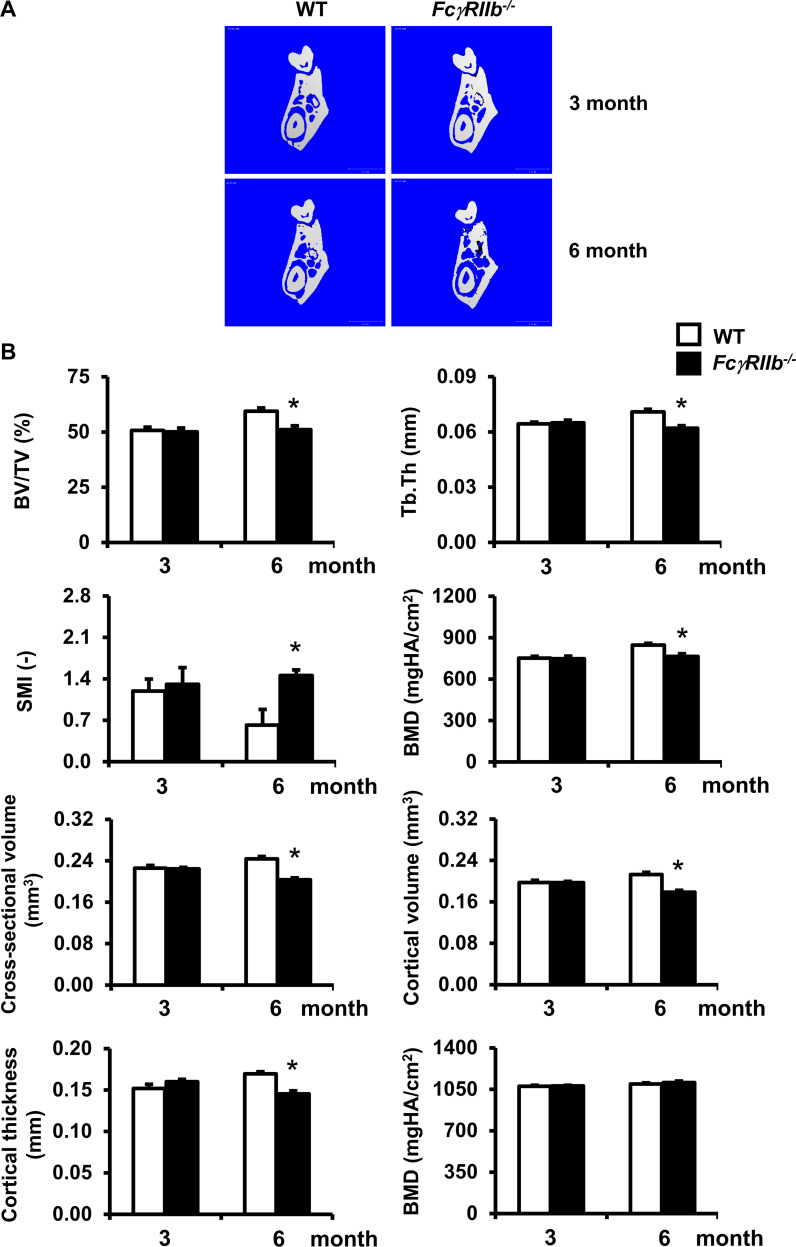
Deletion of *FcγRIIb* induces cancellous and cortical bone loss in mandibles in 6- but not 3-month-old males. (A) Representative μCT images of mandibular bone from 3- and 6-month-old *FcγRIIb*^*-/-*^ mice and WT controls. (B) μCT analysis of cancellous and cortical bone in the mandibles from 3- and 6-month-old *FcγRIIb*^*-/-*^ mice and WT controls. Data are mean ± SEM (n = 7–8). *p < 0.05 versus WT controls.

Previous studies indicated that *FcγRIIb*^*-/-*^ mice developed spontaneous SLE at 6 months of age. *FcγRIIb*^*-/-*^ mice exhibited a normal skeletal phenotype at 3 months of age [12, 15]. To evaluate whether the absence of *FcγRIIb* affected mandibular cancellous and cortical bone in 3-month-old mice, μCT analysis was performed. μCT analysis showed no difference in cancellous bone volume, trabecular thickness, structure model index (SMI), and bone mineral density (BMD) (Fig 1A and 1B). Trabecular number (22.80±1.00 vs 23.38±0.44 /mm) and trabecular separation (0.074±0.001 vs 0.076±0.001 mm) did not change. Cross-sectional volume, cortical volume, and cortical thickness also did not change (Fig 1A and 1B). Marrow volume (0.028±0.002 vs 0.027±0.001 mm^3^) was not altered. These results indicated that *FcγRIIb* deletion did not affect mandibular bone turnover at 3 months of age prior to the development of the lupus phenotype.

To further investigate whether *FcγRIIb* deletion resulted in mandibular abnormalities in mature adult mice with active SLE, we assessed the cancellous and cortical bone microarchitecture of the mandible in 6-month-old *FcγRIIb*^*-/-*^ males. μCT analysis of the mandibles showed that *FcγRIIb*^*-/-*^ mice were osteopenic with significantly decreased cancellous bone volume (11%), and trabecular thickness (10%) (Fig 1B) without any change in trabecular number (20.87±0.50 vs 21.50±0.34 /mm), trabecular separation (0.070±0.001 vs 0.073±0.001 mm), or connectivity density (81±15 vs 102±12 /mm^3^). SMI was increased by 72%. *FcγRIIb*^*-/-*^ mice had lower cancellous BMD (7%) than WT littermate controls, indicating osteopenic phenotype. Cross-sectional volume, cortical volume (Fig 1B), and marrow volume (0.031±0.001 vs 0.025±0.002 mm^3^) were significantly reduced by 15, 14, and 19%, respectively. Cortical thickness was dramatically decreased by 13% (Fig 1B). These findings demonstrated that *FcγRIIb*^*-/-*^ mice had mandibular cancellous and cortical bone loss at 6 months of age.

### TNF-α blockade prevented mandibular bone loss in *FcγRIIb*^*-/-*^ mice

**Fig 2:**
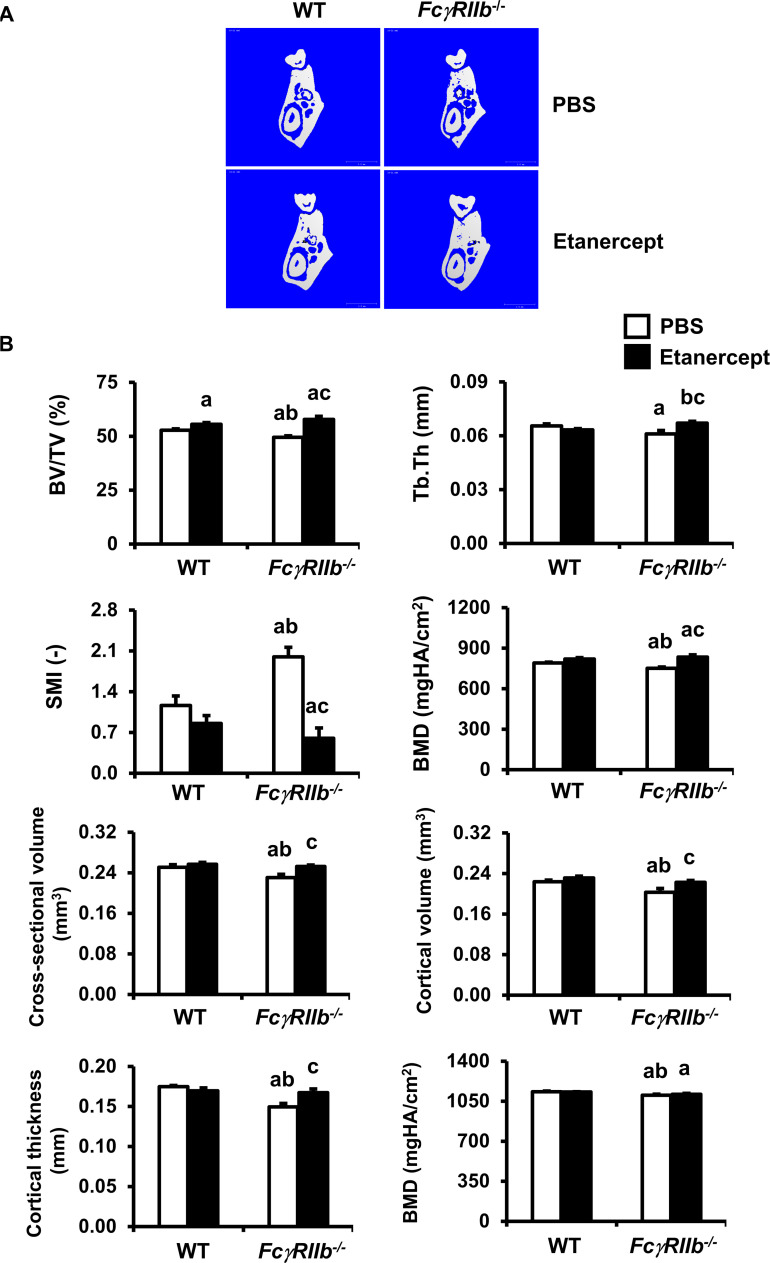
Mandibular cancellous and cortical bone loss are rescued by TNF-α inhibitor in *FcγRIIb*^*-/-*^ mice. (A) Representative μCT images of mandibular bone from *FcγRIIb*^*-/-*^ mice and WT controls after treatment with either PBS or etanercept. (B) μCT analysis of cancellous and cortical bone in the mandibles from *FcγRIIb*^*-/-*^ mice and WT controls after treatment with either PBS or etanercept. Data are mean ± SEM (n = 6–8). ^a^*p* < 0.05 versus WT controls-treated with PBS, ^b^*p* < 0.05 versus WT controls-treated with etanercept, and ^c^*p* < 0.05 versus *FcγRIIb*^-/-^ mice treated with PBS.

Previously we observed that deletion of *FcγRIIb* increased the circulating level of TNF-α at 6 months of age [12]. TNF-α is associated with systemic inflammation in rheumatoid arthritis, ankylosing spondylitis, and periodontitis. Etanercept treatment was used to evaluate whether TNF-α is a key mediator of mandibular bone loss and to test whether TNF-α blockade can reverse this phenotype in the *FcγRIIb*^*-/-*^ lupus model. 6-month-old *FcγRIIb*^*-/-*^ males were treated with etanercept for 8 weeks. Similar to 6-month-old *FcγRIIb*^*-/-*^ mice, 8-month-old *FcγRIIb*^*-/-*^ mice exhibited mandibular bone loss. *FcγRIIb*^*-/-*^ mice had significantly decreased cancellous bone volume, and trabecular thickness (Fig 2A and 2B) without any change in trabecular number (22.20±0.58 vs 22.63±0.51 /mm) and trabecular separation (0.076±0.001 vs 0.072±0.002 mm). SMI was increased in *FcγRIIb*^*-/-*^ mice, indicating a rod like structure. Deletion of *FcγRIIb* decreased cross-sectional volume, cortical volume, and cortical thickness (Fig 2A and 2B), whereas marrow volume did not change (0.027±0.002 vs 0.028±0.001 mm^3^). BMD was decreased in both cortical and cancellous bone (Fig 2B).

μCT analysis showed that TNF-α blockade with etanercept significantly increased cancellous bone volume by 5 and 17% in WT and *FcγRIIb*^*-/-*^ mice, respectively (Fig 2B). Etanercept decreased trabecular separation (0.076±0.001 vs 0.070±0.001 mm) in WT. Trabecular thickness was increased whereas SMI were decreased, indicating plate like structure in *FcγRIIb*^*-/-*^ mice treated with etanercept. Etanercept increased cross-sectional volume, cortical volume, and cortical thickness in *FcγRIIb*^*-/-*^ mice (Fig 2B). Etanercept increased cancellous but not cortical bone mineral density only in *FcγRIIb*^*-/-*^ mice. Two-way ANOVA indicated interaction between *FcγRIIb* deficiency and etanercept on cancellous bone volume, trabecular thickness, SMI, BMD, and cortical thickness. These findings proved that blockade of TNF-α by etanercept could prevent cancellous and cortical bone loss in the mandible.

### TNF-α blockade elevated osteoblast gene expression in *FcγRIIb*^*-/-*^ mice

**Fig 3:**
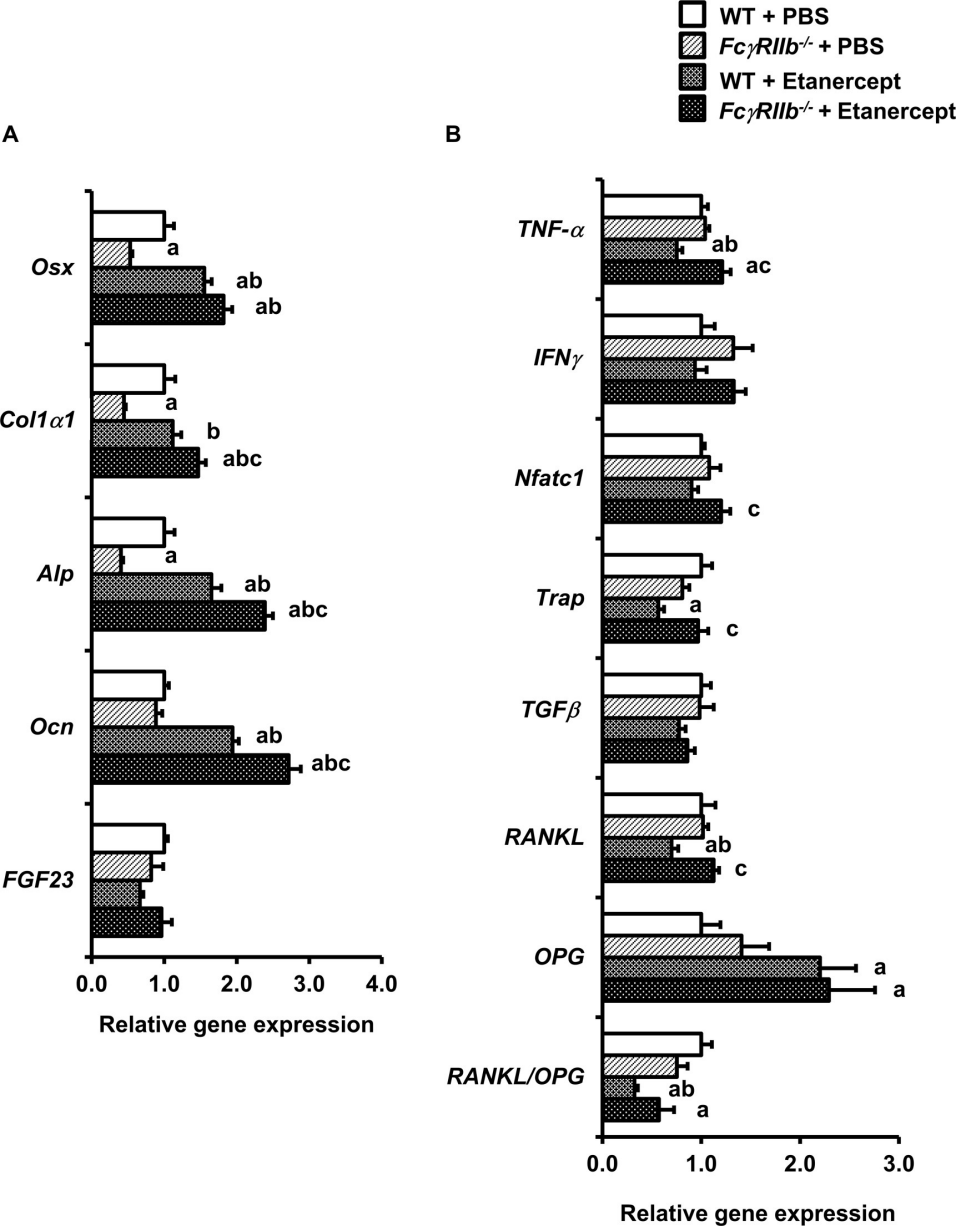
TNF-α blockade upregulates osteoblast marker gene expression in *FcγRIIb*^*-/-*^ mice. (A) qPCR analysis of osteoblast-specific genes. (B) qPCR analysis of osteoclast-specific genes. Data are mean ± SEM (n = 5–6). ^a^*p* < 0.05 versus WT controls-treated with PBS, ^b^*p* < 0.05 versus *FcγRIIb*^-/-^ mice-treated with PBS, and ^c^*p* < 0.05 versus WT controls treated with etanercept.

To better understand the underlying mechanism by which etanercept prevented mandibular bone loss, osteoblast and osteoclast marker gene expression was evaluated. qPCR analysis showed a significant decrease in osteoblast-specific gene expression, including *Osx*, type I collagen α1 (*Col1*α*1*), and *Alp*, whereas *Ocn*, and *FGF23* did not alter in *FcγRIIb*^*-/-*^ mice treated with PBS (Fig 3A). There was no significant difference in a number of genes influencing osteoclastogenesis, including *TNF-*α, *IFNγ*, *Nfatc1*, and *TGFβ* (Fig 3B). Likewise, levels of osteoclast marker gene such as *Trap*, and *RANKL/OPG* were not significant altered. Interestingly, osteoblast-specific genes including *Osx*, *Alp*, and *Ocn* were significantly elevated in WT and *FcγRIIb*^*-/-*^ mice treated with etanercept (Fig 3A). Etanercept increased *Col1a1* expression only in *FcγRIIb*^*-/-*^ mice but not WT. *TNF-*α, *Trap*, *RANKL*, and *RANKL/OPG* ratio were attenuated in WT-treated with etanercept (Fig 3B). However, *IFNγ*, *Nfatc1*, *TGFβ* mRNA levels were not altered. Deletion of *FcγRIIb* did not have any effect on genes related to osteoclastogenesis.

### TNF-α blockade reduced proinflammatory cytokines, and elevated PTH levels in *FcγRIIb*^*-/-*^ mice

**Fig 4:**
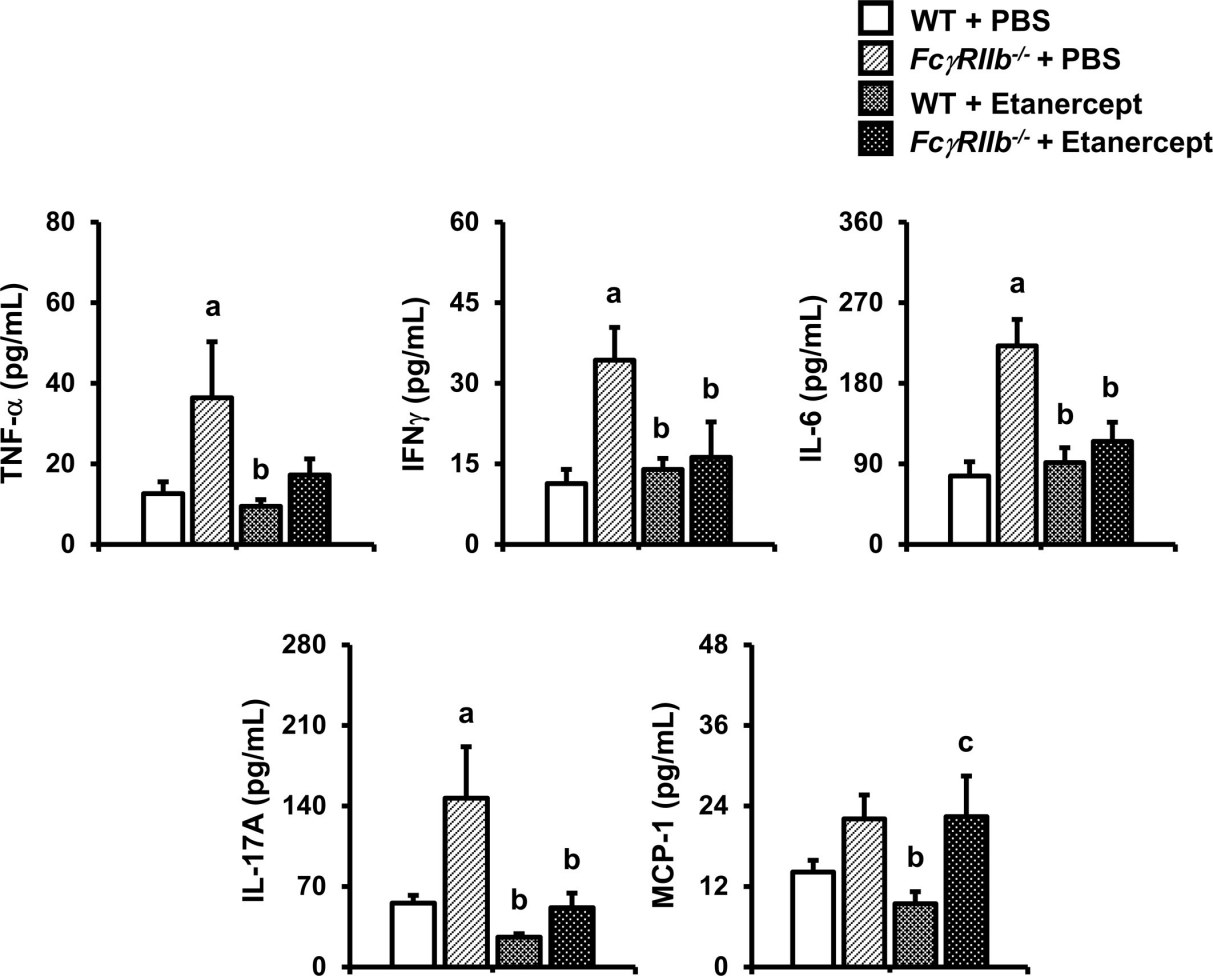
Inflammatory cytokines are reduced by TNF-α antagonist. Serum levels of TNF-α, IFNγ, IL-6, IL-17A, and MCP-1 in *FcγRIIb*^*-/-*^ mice and WT controls after treatment with either PBS or etanercept. Data are mean ± SEM (n = 5–7). ^a^*p* < 0.05 versus WT controls-treated with PBS, ^b^*p* < 0.05 versus *FcγRIIb*^-/-^ mice-treated with PBS, and ^c^*p* < 0.05 versus WT controls treated with etanercept.

TNF-α induced inflammatory bone loss in long bone of *FcγRIIb*^*-/-*^ mice [12]. To further investigate whether etanercept could diminish inflammatory-related mandibular bone loss, the circulating levels of proinflammatory cytokines including IL-23, IL-1α, TNF-α, IFNγ, MCP-1, IL-12p70, IL-1β, IL-10, IL-6, IL-27, IL-17A, IFNβ, GM-CSF were assessed. *FcγRIIb*^*-/-*^ mice had elevated TNF-α, IFNγ, IL-6, IL-17A serum concentrations (Fig 4). Two-way ANOVA confirmed these effects. MCP-1 was slightly increased in *FcγRIIb*^*-/-*^ mice with one-way ANOVA followed by Fisher’s protected least significant difference test (*p* = 0.094) however, two-way ANOVA indicated that deletion of *FcγRIIb* increased MCP-1. Our result confirmed our prior study that the circulating level of TNF-α was elevated following SLE development. IL-1α was decreased in WT-treated with etanercept but other cytokines were not altered (S2 Table). Etanercept slightly decreased serum TNF-α in *FcγRIIb*^*-/-*^ mice (*p* = 0.09). Administration of etanercept significantly decreased serum IFNγ, IL-6 and IL-17A levels in *FcγRIIb*^-/-^ mice. There was no effect on serum IL-23, IL-1α, IL-12p70, IL-1β, IL-10, IL-27, IFNβ, and GM-CSF levels after etanercept treatment (S2 Table). These data suggested that TNF-α inhibition prevented mandibular bone loss by decreasing circulating proinflammatory cytokines in *FcγRIIb*^*-/-*^ mice.

It has been shown that TNF inhibition increases serum PTH levels in patients with rheumatoid arthritis [18]. We determined whether etanercept would increase serum PTH levels in *FcγRIIb*^*-/-*^ mice. Deletion of *FcγRIIb* did not have a significant effect on serum PTH level (77.11±15.82 vs 64.62±13.83 pg/ml). Interestingly, anti-TNF-α treatment increased serum level of PTH only in *FcγRIIb*^*-/-*^ mice (64.62±13.83 vs 113.31±15.44 pg/ml, *p*<0.05) but not WT (77.11±15.82 vs 54.70±6.07 pg/ml). Two-way ANOVA indicated interaction between *FcγRIIb* deficiency and etanercept on serum PTH.

## Discussion

SLE is a chronic auto-immune inflammatory disease characterized by loss of self-tolerance with activation of autoreactive T and B cells resulting in hyperproduction of autoantibodies and damage to multiple organs. SLE patients are likely to suffer from periodontitis and SLE shares common pathogenic inflammatory characteristics with periodontitis. Oral manifestations of patients with SLE are common and typically include oral lesions coinciding with disease flares that lead to alveolar bone loss [19]. SLE patients have oral ulceration, honeycomb plaque, purpura, and petechiae in oral mucous membrane and periodontal tissue. TNF-α-induced inflammation may contribute to severe periodontitis leading to alveolar bone loss in SLE patients. We previously reported that absence of *FcγRIIb* resulted in spontaneous development of active SLE with overproduction of anti-dsDNA antibodies, increased splenic B220^low^CD138^+^ plasma cells, and induced osteopenia in the long bones of male mice [12, 15]. However, there has been a lack of clinical data characterizing the effects of the inhibitory *FcγR*, *FcγRIIb*, on mandibular bone homeostasis in SLE patients with periodontitis.

The present study aimed to investigate whether TNF-α is a key mediator of mandibular bone loss in the *FcγRIIb*^*-/-*^ model of lupus. Our data indicated that genetic deficiency of *FcγRIIb* induced mandibular bone loss in both cancellous and cortical bone sites by increasing proinflammatory cytokines and that TNF-α antagonist prevented proinflammatory cytokines-mediated mandibular bone loss in *FcγRIIb*^*-/-*^ mice. Increases in cancellous and cortical bone volume were associated with increased bone formation and decreased bone resorption in WT-treated with etanercept. The expression of osteoblast marker genes, *Osx*, *Alp* and *Ocn*, were upregulated whereas osteoclast marker genes, *TNF-*α, *Trap*, *RANKL/OPG* ratio, were downregulated. However, etanercept increased *Osx*, *Col1a1*, *Alp* and *Ocn* without any alteration in osteoclast specific genes in *FcγRIIb*^*-/-*^ mice. Deletion of *FcγRIIb* increased proinflammatory cytokines, including TNF-α, IFNγ, IL6, and IL-17A, and blocking TNF-α blunted these effects.

Genetic polymorphisms for FcγR, a member of immunoglobulin superfamily, were found in both SLE and periodontitis [14]. *FcγRIIb-232T* less effectively inhibited B cell receptor-mediated activation signal than *FcγRIIb-232I*, resulting in hyperactivation of B cells and increased risk for autoimmunity [20]. Kobayashi et al. demonstrated that both the stimulatory *FcγRIIa-R131* and the inhibitory *FcγRIIb-232T* alleles were associated with SLE and periodontitis [14]. A combination of both *FcγR* risk alleles was associated with more severe periodontitis. *FcγRI* levels were upregulated whereas *FcγRIIa* and *FcγRIIIb* were downregulated in gingival crevicular fluid PMN [21]. The increased *FcγRI* expression by cytokines and bacterial stimuli concomitantly elevated IgA-mediated anti-*P*. *gingivalis* function, leading to clearance of *P*. *gingivalis*.

Both MRL/*lpr* and BXSB/MpJ-Yaa mice are well-established mouse models of SLE-related osteoporosis. A study by Schapira et al. reported that MRL/*lpr* mice with SLE-like phenotypes had decreased bone formation [22]. Transplantation of human bone marrow mesenchymal stem cells and stem cells from exfoliated deciduous teeth into MRL/*lpr* mice ameliorated severe bone loss by increasing osteoblastogenesis and decreasing osteoclastogenesis [23]. Three-month-old BXSB/MpJ-Yaa mice carrying a Y-linked autoimmune acceleration gene (Yaa) exhibited a normal bone homeostasis. However, osteopenia was observed at 6 months of age due to increased bone resorption with normal bone formation [24]. This study suggests that our finding of mandibular bone loss in *FcγRIIb*^*-/-*^ lupus mice is likely broadly applicable to other SLE models.

In SLE patients, proinflammatory cytokines drive uncoupling of bone formation and bone resorption, resulting in osteopenia [25]. The underlying mechanisms of TNF-α-mediated bone loss in *FcγRIIb* deficiency were not fully understood. We observed that in *FcγRIIb*^*-/-*^ mice elevations in serum TNF-α were associated with decreases in the expression of osteoblast-specific genes, including *Osx*, *Col1a1*, and *Alp*. This finding indicated that attenuations in mandibular cancellous and cortical bone volume were due to reduced bone formation in *FcγRIIb*^*-/-*^ mice. The use of anti-TNF-α therapy in SLE is controversial. Anti-TNF-α drugs suppresses the local tissue damage, however the use of this drug is associated with the formation of autoantibodies, including antinuclear antibodies, antidouble-stranded DNA antibodies, and anticardiolipin antibodies [26]. As a consequence, TNF-α blockade could pose long-term danger in patients with lupus nephritis and symptoms can recur after cessation of anti-TNF-α therapy. TNF-α inhibitor treatment decreased staphylococcal enterotoxin-induced inflammatory arthritis, autoantibody formation and serum TNF-α production in MRL/*lpr* mice [27] We found that mandibular cortical and cancellous bone loss were rescued in *FcγRIIb*^*-/-*^ mice administered with etanercept. Etanercept significantly upregulated the expression of osteoblast specific genes including *Osx*, *Alp*, and *Ocn* expression, and downregulated the expression of genes influencing osteoclastogenesis such as *TNF-*α, *Trap*, RANKL*/OPG* ratio in WT. However, *Osx*, *Col1a1*, *Alp*, and *Ocn* were increased in *FcγRIIb*^*-/-*^ mice-treated with etanercept without any change in osteoclast marker genes.

Imbalance between activating and inhibitory *FcγR* functions disposes individuals to autoimmune inflammatory disease. *FcγRIIb* inhibition promotes the overproduction of pro-inflammatory cytokines, including TNF-α which is active in inflammatory diseases, including Grave’s disease, rheumatoid arthritis, periodontitis and SLE [28]. In periodontitis, TNF-α was involved in immunoregulatory and inflammatory processes that recruits neutrophils and other leukocytes to the inflammation site. Elevations in TNF-α serum level were found in both SLE mouse models and in lupus patients with periodontitis, and these elevations positively correlated with disease activity [12, 25, 29]. We observed that *FcγRIIb*-deficient males had dramatically increased serum levels of TNF-α, IFNγ, IL-6, and IL-17A, whereas level of IL-23, IL-1α, IL-12p70, IL-1β, IL-10, IL-27, IFNβ, and GM-CSF remained unchanged. MCP-1 production was slightly increased in *FcγRIIb*^*-/-*^ mice. These findings were consistent with clinical investigations in SLE patients, since increased expression of TNF-α, IFNγ, IL-6, and IL-17A had been documented [13, 30, 31]. SLE patients had a worse periodontitis due to high IFNγ levels [2]. Salivary concentrations of IL-6, and IL-17A were increased in SLE patients with periodontitis [31]. Our results showed a similar alteration of proinflammatory cytokines associated with mandibular bone loss in the absence of *FcγRIIb*.

Cytokines are involved in osteoimmunology and inflammatory bone diseases. An increase in proinflammatory cytokines may contribute to dysregulation of *RANKL/OPG* cascades resulting in enhanced osteoclastogenesis. Deletion of *FcγRIIb* increased proinflammatory cytokines, TNF-α, IFNγ, IL-6, and IL-17A, that mediated cancellous and cortical bone loss. Bone destruction is accelerated in inflammatory diseases associated with increased TNF-α production, including ankylosing spondylitis, inflammatory bowel disease, periodontitis and rheumatoid arthritis. TNF-α stimulates bone resorption by acting directly on osteoclast precursors to promote osteoclastogenesis. It also acts on osteoblast to induce RANKL production. High serum levels of TNF-α and IL-6 in patients with SLE have been reported [32]. IL-17A induces bone resorption downstream of TNF-α. IL-17A blockade attenuates TNF-α-mediated inflammatory arthritis and bone loss in transgenic mice [33]. The role of IFNγ in the regulation of bone formation and bone resorption remains controversial. A study of Duque et al, indicated that IFNγ increased cancellous bone volume by increasing osteoblast and osteoclast number in both sham and ovariectomized (OVX) mice [34]. In contrast, IFNγ receptor knockout mice are osteopenic due to decreased bone formation and bone resorption. Mice with silencing of IFNγ receptor signaling are protected from OVX-induced bone loss [35] Others report that while IFNγ has a direct action to inhibit osteoclast formation by osteoclast precursors, it also has indirect actions to stimulate osteoclastogenesis through stimulating RANKL expression and TNF-α secretion from T cell [36].

TNF inhibitor increased serum PTH level in patients with rheumatoid arthritis [18]. Anti-TNF-α therapy in Crohn’s disease, a disorder characterized by overproduction of TNF-α resulted in higher levels of PTH [37]. Our findings are consistent with these studies finding that serum PTH was elevated following etanercept administration. We speculated that the increased bone formation after anti-TNF-α treatment might be driven at least in part by this increase in PTH levels. *TNF-*α, *Trap*, *RANKL/OPG* ratio, makers of bone resorption, were decreased in WT-treated with etanercept, indicating reduced bone resorption. However, we did not observe any change in bone resorption in *FcγRIIb*^*-/-*^ mice after anti-TNF-α treatment, possibly due to high serum level of PTH.

## Conclusions

Six months old mice lacking *FcγRIIb* developed SLE and were susceptible to TNF-α-induced inflammatory bone loss in the mandible. Moreover, *FcγRIIb*^-/-^ mice treated with etanercept were strongly protected from TNF-α-mediated mandibular bone loss. Absence of *FcγRIIb* increased bone formation and decreased inflammatory cytokines after administration of a TNF-α blocker. Our recent findings suggest that blockade of TNF-α may be beneficial for periodontal diseases in SLE patients.

## Supporting information

S1 Table List of mouse primer sequences for qPCR analysis. (PDF)

S2 Table Serum proinflammatory cytokines in *FcγRIIb^-/-^* males and their control littermates treated with either PBS or etanercept. (PDF)

## References

[1] Bagavant H, Dunkleberger ML, Wolska N, Sroka M, Rasmussen A, Adrianto I, et al. Antibodies to periodontogenic bacteria are associated with higher disease activity in lupus patients. Clin Exp Rheumatol. 2019;37(1):106–11. 29998833 PMC6309750

[2] Sete MRC, Carlos JC, Lira-Junior R, Boström EA, Sztajnbok FR, Figueredo CM. Clinical, immunological and microbial gingival profile of juvenile systemic lupus erythematosus patients. Lupus. 2019;28(2):189–98. 10.1177/0961203318819134 30563424

[3] Fredman G, Oh SF, Ayilavarapu S, Hasturk H, Serhan CN, Van Dyke TE. Impaired Phagocytosis in Localized Aggressive Periodontitis: Rescue by Resolvin E1. PLOS ONE. 2011;6(9):e24422. 10.1371/journal.pone.0024422 21935407 PMC3173372

[4] Nimmerjahn F, Ravetch JV. Fcgamma receptors: old friends and new family members. Immunity. 2006;24(1):19–28. 10.1016/j.immuni.2005.11.010 16413920

[5] Nicu EA, Van der Velden U, Everts V, Van Winkelhoff AJ, Roos D, Loos BG. Hyper-reactive PMNs in FcgammaRIIa 131 H/H genotype periodontitis patients. Journal of clinical periodontology. 2007;34(11):938–45. 10.1111/j.1600-051X.2007.01136.x 17877745

[6] Rhodus NL, Johnson DK. The prevalence of oral manifestations of systemic lupus erythematosus. Quintessence international (Berlin, Germany: 1985). 1990;21(6):461–5. 2243950

[7] Bolland S, Ravetch JV. Spontaneous autoimmune disease in Fc(gamma)RIIB-deficient mice results from strain-specific epistasis. Immunity. 2000;13(2):277–85. 10.1016/s1074-7613(00)00027-3 10981970

[8] McGaha TL, Sorrentino B, Ravetch JV. Restoration of tolerance in lupus by targeted inhibitory receptor expression. Science. 2005;307(5709):590–3. 10.1126/science.1105160 15681388

[9] Yuasa T, Kubo S, Yoshino T, Ujike A, Matsumura K, Ono M, et al. Deletion of fcgamma receptor IIB renders H-2(b) mice susceptible to collagen-induced arthritis. The Journal of experimental medicine. 1999;189(1):187–94. 10.1084/jem.189.1.187 9874575 PMC1887699

[10] Jacobi AM, Mei H, Hoyer BF, Mumtaz IM, Thiele K, Radbruch A, et al. HLA-DRhigh/CD27high plasmablasts indicate active disease in patients with systemic lupus erythematosus. Ann Rheum Dis. 2010;69(1):305–8. 10.1136/ard.2008.096495 19196727

[11] Odendahl M, Jacobi A, Hansen A, Feist E, Hiepe F, Burmester GR, et al. Disturbed peripheral B lymphocyte homeostasis in systemic lupus erythematosus. Journal of immunology (Baltimore, Md: 1950). 2000;165(10):5970–9. 10.4049/jimmunol.165.10.5970 11067960

[12] Visitchanakun P, Saiworn W, Jongwattanapisan P, Leelahavanichkul A, Pisitkun P, Lotinun S. Lupus-like Disease in FcγRIIB−/− Mice Induces Osteopenia. Sci Rep. 2019;9(1):17342. 10.1038/s41598-019-53963-z 31758072 PMC6874658

[13] Gumus P, Nizam N, Lappin DF, Buduneli N. Saliva and serum levels of B-cell activating factors and tumor necrosis factor-alpha in patients with periodontitis. Journal of periodontology. 2014;85(2):270–80. 10.1902/jop.2013.130117 23701482

[14] Kobayashi T, Ito S, Yasuda K, Kuroda T, Yamamoto K, Sugita N, et al. The combined genotypes of stimulatory and inhibitory Fc gamma receptors associated with systemic lupus erythematosus and periodontitis in Japanese adults. Journal of periodontology. 2007;78(3):467–74. 10.1902/jop.2007.060194 17335370

[15] Saiworn W, Thim-Uam A, Visitchanakun P, Atjanasuppat K, Chantaraaumporn J, Mokdara J, et al. Cortical Bone Loss in a Spontaneous Murine Model of Systemic Lupus Erythematosus. Calcified tissue international. 2018;103(6):686–97. 10.1007/s00223-018-0464-7 30116830

[16] Yoshitaka T, Ishida S, Mukai T, Kittaka M, Reichenberger EJ, Ueki Y. Etanercept administration to neonatal SH3BP2 knock-in cherubism mice prevents TNF-α-induced inflammation and bone loss. Journal of bone and mineral research: the official journal of the American Society for Bone and Mineral Research. 2014;29(5):1170–82. 10.1002/jbmr.2125 PMC4131552 24978678

[17] Bouxsein ML, Boyd SK, Christiansen BA, Guldberg RE, Jepsen KJ, Müller R. Guidelines for assessment of bone microstructure in rodents using micro-computed tomography. Journal of bone and mineral research: the official journal of the American Society for Bone and Mineral Research. 2010;25(7):1468–86. 10.1002/jbmr.141 20533309

[18] Adami G, Orsolini G, Adami S, Viapiana O, Idolazzi L, Gatti D, et al. Effects of TNF Inhibitors on Parathyroid Hormone and Wnt Signaling Antagonists in Rheumatoid Arthritis. Calcified tissue international. 2016;99(4):360–4. 10.1007/s00223-016-0161-3 27307275

[19] Aringer M, Johnson SR. Classifying and diagnosing systemic lupus erythematosus in the 21st century. Rheumatology (Oxford). 2020;59(Supplement_5):v4–v11. 10.1093/rheumatology/keaa379 33280013 PMC7719035

[20] Kono H, Kyogoku C, Suzuki T, Tsuchiya N, Honda H, Yamamoto K, et al. FcgammaRIIB Ile232Thr transmembrane polymorphism associated with human systemic lupus erythematosus decreases affinity to lipid rafts and attenuates inhibitory effects on B cell receptor signaling. Human molecular genetics. 2005;14(19):2881–92. 10.1093/hmg/ddi320 16115811

[21] Kobayashi T, Yamamoto K, Sugita N, van Spriel AB, Kaneko S, van de Winkel JG, et al. Effective in vitro clearance of Porphyromonas gingivalis by Fc alpha receptor I (CD89) on gingival crevicular neutrophils. Infect Immun. 2001;69(5):2935–42. 10.1128/IAI.69.5.2935-2942.2001 11292709 PMC98245

[22] Schapira D, Kabala A, Raz B, Israeli E. Osteoporosis in murine systemic lupus erythematosus—a laboratory model. Lupus. 2001;10(6):431–8. 10.1191/096120301678646182 11434579

[23] Ma L, Aijima R, Hoshino Y, Yamaza H, Tomoda E, Tanaka Y, et al. Transplantation of mesenchymal stem cells ameliorates secondary osteoporosis through interleukin-17-impaired functions of recipient bone marrow mesenchymal stem cells in MRL/lpr mice. Stem Cell Res Ther. 2015;6:104. 10.1186/s13287-015-0091-4 26012584 PMC4474573

[24] Namba T, Ichii O, Nakamura T, Masum MA, Otani Y, Otsuka-Kanazawa S, et al. Feature Article: Altered morpho-functional features of bones in autoimmune disease-prone BXSB/MpJ- Yaa mice. Experimental biology and medicine (Maywood, NJ). 2019;244(5):333–43. 10.1177/1535370219832810 30818998 PMC6488867

[25] Amarasekara DS, Yu J, Rho J. Bone Loss Triggered by the Cytokine Network in Inflammatory Autoimmune Diseases. Journal of immunology research. 2015;2015:832127. 10.1155/2015/832127 26065006 PMC4434203

[26] Zhu LJ, Yang X, Yu XQ. Anti-TNF-alpha therapies in systemic lupus erythematosus. J Biomed Biotechnol. 2010;2010:465898. 10.1155/2010/465898 20625488 PMC2896679

[27] Edwards CK 3rd, Zhou T, Zhang J, Baker TJ, De M, Long RE, et al. Inhibition of superantigen-induced proinflammatory cytokine production and inflammatory arthritis in MRL-lpr/lpr mice by a transcriptional inhibitor of TNF-alpha. Journal of immunology (Baltimore, Md: 1950). 1996;157(4):1758–72. 8759766

[28] Vogelpoel L, Baeten D, de Jong E, Den Dunnen J. Control of cytokine production by human Fc gamma receptors: implications for pathogen defense and autoimmunity. Frontiers in immunology. 2015;6(79). 10.3389/fimmu.2015.00079 25759693 PMC4338787

[29] Varghese SS, Thomas H, Jayakumar ND, Sankari M, Lakshmanan R. Estimation of salivary tumor necrosis factor-alpha in chronic and aggressive periodontitis patients. Contemporary clinical dentistry. 2015;6(Suppl 1):S152–6. 10.4103/0976-237X.166816 26604566 PMC4632215

[30] Pessoa L, Aleti G, Choudhury S, Nguyen D, Yaskell T, Zhang Y, et al. Host-Microbial Interactions in Systemic Lupus Erythematosus and Periodontitis. Frontiers in immunology. 2019;10:2602. 10.3389/fimmu.2019.02602 31781106 PMC6861327

[31] Mendonça SMS, Corrêa JD, Souza AF, Travassos DV, Calderaro DC, Rocha NP, et al. Immunological signatures in saliva of systemic lupus erythematosus patients: influence of periodontal condition. Clin Exp Rheumatol. 2019;37(2):208–14. 30148445

[32] Umare V, Pradhan V, Nadkar M, Rajadhyaksha A, Patwardhan M, Ghosh KK, et al. Effect of proinflammatory cytokines (IL-6, TNF-alpha, and IL-1beta) on clinical manifestations in Indian SLE patients. Mediators Inflamm. 2014;2014:385297. 10.1155/2014/385297 25548434 PMC4273527

[33] Zwerina K, Koenders M, Hueber A, Marijnissen RJ, Baum W, Heiland GR, et al. Anti IL-17A therapy inhibits bone loss in TNF-alpha-mediated murine arthritis by modulation of the T-cell balance. Eur J Immunol. 2012;42(2):413–23. 10.1002/eji.201141871 22101928

[34] Duque G, Huang DC, Dion N, Macoritto M, Rivas D, Li W, et al. Interferon-γ plays a role in bone formation in vivo and rescues osteoporosis in ovariectomized mice. Journal of bone and mineral research: the official journal of the American Society for Bone and Mineral Research. 2011;26(7):1472–83. 10.1002/jbmr.350 21308779

[35] Cenci S, Toraldo G, Weitzmann MN, Roggia C, Gao Y, Qian WP, et al. Estrogen deficiency induces bone loss by increasing T cell proliferation and lifespan through IFN-gamma-induced class II transactivator. Proceedings of the National Academy of Sciences of the United States of America. 2003;100(18):10405–10. 10.1073/pnas.1533207100 12923292 PMC193574

[36] Gao Y, Grassi F, Ryan MR, Terauchi M, Page K, Yang X, et al. IFN-gamma stimulates osteoclast formation and bone loss in vivo via antigen-driven T cell activation. The Journal of clinical investigation. 2007;117(1):122–32. 10.1172/JCI30074 17173138 PMC1697800

[37] Augustine MV, Leonard MB, Thayu M, Baldassano RN, de Boer IH, Shults J, et al. Changes in vitamin D-related mineral metabolism after induction with anti-tumor necrosis factor-alpha therapy in Crohn’s disease. J Clin Endocrinol Metab. 2014;99(6):E991–8. 10.1210/jc.2013-3846 24617709 PMC4037735

