# Supplementary material for: Etanercept prevents TNF-α mediated mandibular bone loss in FcγRIIb-/- lupus model
Source: PLoS One. 2021 Apr 16;16(4):e0250215. doi: 10.1371/journal.pone.0250215 (PMC8051757; doi:10.1371/journal.pone.0250215)
Supplement: S1 Table — (PDF) [file pone.0250215.s001.pdf]

S1 Table. List of mouse primer sequences for qPCR analysis.

Gene	Forward sequences	Reverse sequences
Osx	CCCTTCTCAAGCACCAATGG	AAGGGTGGGTAGTCATTTGCATA
Col1a1	CCCAAGGAAAAGAAGCACGTC	ACATTAGGCGCAGGAAGGTCA
Alp	CTTGACTGTGGTTACTGCTGATCA	GTATCCACCGAATGTGAAAACGT
Ocn	GCTGCCCTAAAGCCAAACTCT	AGAGGACAGGGAGGATCAAGTTC
FGF23	AGGACCAGCTATCACCTACA	CGAGTCATGGCTCCTGTTATC
Tnfα	TTGTCTACTCCCAGGTTCTCT	GAGGTTGACTTTCTCCTGGTATG
IFNγ	AAATCCTGCAGAGCCAGATTAT	GCTGTTGCTGAAGAAGGTAGTA
Nfatc1	AGGCTGGTCTTCCGAGTTCA	ACCGCTGGGAACACTCGAT
Trap	GATCCCTCTGTGCGACATCA	CCAGGGAGTCCTCAGATCCA
TGFβ	GGTGGTATACTGAGACACCTTG	CCCAAGGAAAGGTAGGTGATAG
RANKL	CAAGCTCCGAGCTGGTGAAG	CCTGAACTTTGAAAGCCCCA
OPG	AAGAGCAAACCTTCCAGCTGC	CACGCTGCTTTCACAGAGGTC
GAPDH	TGCACCACCAACTGCTTAG	GGATGCAGGGATGATGTTC
