# Supplementary material for: Etanercept prevents TNF-α mediated mandibular bone loss in FcγRIIb-/- lupus model
Source: PLoS One. 2021 Apr 16;16(4):e0250215. doi: 10.1371/journal.pone.0250215 (PMC8051757; doi:10.1371/journal.pone.0250215)
Supplement: S2 Table — (PDF) [file pone.0250215.s002.pdf]

S2 Table. Serum proinflammatory cytokines in *FcγRIIb*$^{-/-}$ males and their control littermates treated with either PBS or etanercept.

Parameters	PBS		Etanercept		Two-way ANOVA		
	WT (n=7)	*FcγRIIb*$^{-/-}$ (n=6)	WT (n=7)	*FcγRIIb*$^{-/-}$ (n=5)	*FcγRIIb*$^{-/-}$	Etanercept	Interaction
IL-23 (pg/ml)	31.64±10.12	30.04±7.06	24.57±6.34	23.89±10.13	NS	NS	NS
IL-1α (pg/ml)	7.03±0.79	5.75±1.36	3.72±0.54[a]	6.59±1.29	NS	NS	$p < 0.05$
TNF-α (pg/ml)	12.59±2.95	36.42±13.90[a]	9.44±1.67[b]	17.23±4.01	$p < 0.05$	NS	NS
IFNγ (pg/ml)	11.31±2.66	34.31±6.09[a]	13.97±2.03[b]	16.20±6.60[b]	$p < 0.05$	NS	$p < 0.05$
MCP-1 (pg/ml)	14.16±1.75	22.06±3.56	9.42±1.81[b]	22.40±6.06[c]	$p < 0.05$	NS	NS
IL-12p70 (pg/ml)	9.89±3.02	12.78±3.66	7.03±0.92	12.64±4.59	NS	NS	NS
IL-1β (pg/ml)	59.18±20.37	70.89±20.71	60.07±13.42	53.04±11.18	NS	NS	NS
IL-10 (pg/ml)	664±121	675±104	574±61	519±143	NS	NS	NS
IL-6 (pg/ml)	76.46±15.82	221.81±29.42[a]	91.34±16.57[b]	115.06±21.40[b]	$p < 0.05$	$p < 0.05$	$p < 0.05$
IL-27 (pg/ml)	535±298	438±180	166±74	283±141	NS	NS	NS
IL-17A (pg/ml)	55.56±6.68	146.73±44.78[a]	25.90±2.98[b]	51.55±12.70[b]	$p < 0.05$	$p < 0.05$	NS
IFNβ (pg/ml)	33.85±9.46	39.58±11.01	26.21±8.32	21.58±9.23	NS	NS	NS
GM-CSF (pg/ml)	23.93±2.97	35.65±10.63	35.93±10.16	49.29±20.22	NS	NS	NS

[a]$p<0.05$ versus WT controls treated with PBS, One-way ANOVA followed by Fisher's PLSD.
[b]$p<0.05$ versus *FcγRIIb*$^{-/-}$ mice treated with PBS.
[c]$p<0.05$ versus WT controls treated with etanercept.
